# Beyond Mean Absolute Relative Difference (MARD): Rethinking Continuous Glucose Monitoring (CGM) Evaluation for Real-World Diabetes Care

**DOI:** 10.7759/cureus.91427

**Published:** 2025-09-01

**Authors:** Matteo Acanfora, Erika Pedone, Barbara Presciuttini, Francesca Ré, Luca Acanfora

**Affiliations:** 1 Institute of Endocrine and Metabolic Sciences, Istituto di Ricovero e Cura a Carattere Scientifico (IRCCS) Ospedale San Raffaele, Milan, ITA; 2 Endocrinology Unit, Istituto di Ricovero e Cura a Carattere Scientifico (IRCCS) Ospedale San Raffaele, Vita-Salute San Raffaele University, Milan, ITA; 3 Diabetology Unit, Istituto di Ricovero e Cura a Carattere Scientifico (IRCCS) Ospedale San Raffaele, Milan, ITA; 4 Endocrinology Unit, Azienda Socio Sanitaria Territoriale (ASST) Mantova, Mantua, ITA; 5 Department of General Surgery, Villa Scassi Hospital, Genoa, ITA; 6 Department of Internal Medicine, Civil Hospital of Ovada, Ovada, ITA

**Keywords:** continuous glucose monitoring (cgm), endocrinology and diabetes, mard, measurement accuracy, safety study

## Abstract

Continuous glucose monitoring (CGM) has transformed diabetes mellitus management, evolving from a supportive monitoring tool to a central pillar of care. For people with type 1 diabetes and many insulin-treated individuals with type 2 diabetes, CGM now directly informs treatment decisions, especially when integrated with automated insulin delivery (AID) systems. In these hybrid closed-loop systems, sensor data drives real-time insulin adjustments, meaning that accuracy is not just a matter of measurement quality; it is a matter of patient safety. However, the primary accuracy measure currently used, the mean absolute relative difference (MARD), is increasingly inadequate for guiding clinical decisions. MARD offers a single averaged number under controlled conditions, but it does not capture the timing, direction, or clinical consequences of sensor errors. This is particularly problematic in AID systems, where even minor inaccuracies may lead to inappropriate insulin dosing, increasing the risk of hypoglycemia or hyperglycemia. Given the centrality of CGM in modern diabetes care, a more comprehensive evaluation approach is urgently needed, one that reflects real-world performance, prioritizes patient safety, and addresses the diverse contexts in which CGM devices are used. This editorial presents an opinion-based perspective, proposing a four-dimensional framework for CGM evaluation beyond the traditional reliance on MARD.

## Editorial

Continuous glucose monitoring (CGM) systems have become indispensable in the management of type 1 diabetes and are increasingly used in insulin-treated type 2 diabetes. While the mean absolute relative difference (MARD) remains the standardly used index of sensor accuracy, its limitations are well recognized. MARD originated as a simple statistical index in early validation studies of CGM devices, which is the average percentage error between CGM readings and a reference blood glucose value (e.g., capillary glucometer, venous plasma glucose, or laboratory assays). However, MARD is typically derived under ideal laboratory conditions and does not capture the timing, direction, or clinical consequences of sensor deviations [1].

In automated insulin delivery (AID) and hybrid closed-loop (HCL) systems, even slight inaccuracies can lead to inappropriate insulin dosing decisions. Undetected nocturnal hypoglycemia, overestimated postprandial values, and delayed detection of exercise-induced hypoglycemia have all been documented as safety risks [1].

We propose moving beyond single-number metrics and adopting a four-dimensional evaluation framework that encompasses safety, performance, usability, and equity (which are largely interdependent), dimensions that cannot be adequately captured by any single metric, which together ensure that no critical aspect is overlooked (see CGM metrics in Table 1). Safety reflects the ability to prevent harmful errors such as missed hypoglycemia; performance relates to overall analytical accuracy across glucose ranges; usability addresses patient experience and integration into daily life; and equity ensures that benefits are accessible across diverse populations. Together, these four pillars may provide a more balanced assessment of CGM than MARD alone, or at the very least foster a constructive debate aimed at overcoming current limitations.

**Table 1:** Classical and proposed CGM metrics. Summary of classical and emerging continuous glucose monitoring (CGM) metrics. MARD (Mean Absolute Relative Difference), GRI (Glycemic Risk Index), SEG (Surveillance Error Grid), and PARD (Precision Absolute Relative Difference) are used to assess accuracy and clinical risk. At the same time, TIR (Time in Range) measures time within the target glucose range. Factory-calibrated CGM systems, along with optional user calibration, address device accuracy under various conditions. Usability metrics capture patient-reported ease of use and satisfaction with the system. T1D: type 1 diabetes; T2D: type 2 diabetes; SBGM: self blood glucose monitoring (with a glucometer).

Metric	Definition	Strengths	Limitations
MARD	Mean absolute relative difference between CGM readings and a reference	Easy to calculate; historical benchmark	Ignores timing, direction, and clinical consequences
GRI	Composite index quantifying glycemic risk	Integrates accuracy and clinical impact	Less familiar; more complex to calculate
SEG	Grid-based classification of errors by clinical severity	Provides clinical context for errors	Requires detailed data and validation
PARD	Point-by-point precision, absolute and relative difference	Sensitive to short-term variations	Lacks temporal and clinical context
TIR	Percentage of time in target glucose range (at least 70% of readings between 70 and 180 mg/dL [3.9–10 mmol/L] for adults with T1D or T2D)	Strongly linked to long-term clinical outcomes	Does not measure accuracy directly
Factory-calibrated CGM	Pre-calibrated by the manufacturer	Reduces user error, improves consistency	Cost and technology considerations
Optional user calibration (SBGM)	Manual calibration with capillary glucose when needed	Restores accuracy in specific circumstances; boosts user confidence	Requires correct technique and timing
Usability metrics	Patient-reported ease of use and experience	Direct impact on adherence and satisfaction	Subjective and population-dependent

Safety

Beyond MARD, other indices, introduced through academic and clinical research, include the Glycemic Risk Index (GRI), which provides a composite score of hypo- and hyperglycemia risk; the Surveillance Error Grid (SEG), which maps measurement errors to graded clinical risk zones; and the Precision Absolute Relative Difference (PARD), which captures the dispersion of accuracy. Additionally, hypoglycemia detection rates are recognized as a pragmatic measure of safety. These metrics are derived automatically from CGM datasets, with algorithm development informed by expert input and methodological validation in clinical studies. Routine calculation is fully automated once the algorithm is in place. Despite their stronger clinical meaning, these metrics remain clinically underused because regulatory standards, ISO 15197:2013 (International Organization for Standardization), CLSI (Clinical and Laboratory Standards Institute), FDA (U.S. Food and Drug Administration), and EMA (European Medicines Agency), have maintained MARD as the minimum required metric for CGM commercialization, likely for historical reasons and inertia, as well as because it is easy to calculate and straightforward to communicate as an average percentage error [1-4].

Performance

Time in range (TIR) and glycated hemoglobin (HbA1c) improvements have a strong evidence base linking them to reduced risk of microvascular and macrovascular complications [5]. Performance and safety are interdependent with each other and with the metrics mentioned above. Moreover, modern factory-calibrated CGMs, pre-calibrated by the manufacturer, have demonstrated improved consistency and reduced user-dependent variability in real-world use. Some models also allow optional user-initiated calibration using a capillary glucose measurement through self blood glucose monitoring (SBGM). This feature can be valuable for restoring accuracy in situations such as the physiological lag between blood and interstitial glucose, sensor compression effects (e.g., during sleep), reduced accuracy during the early wear-in period with progressive improvement after the first day, altered tissue perfusion, drug interferences (e.g., acetaminophen, ascorbic acid), calibration issues (e.g., errors from inaccurate fingerstick references or calibrating during rapid glucose changes), and sensor drift (progressive loss of accuracy over time), thus providing an additional safeguard in daily management [6].

Usability

User-friendly design and reduction of alarm fatigue are crucial determinants of adherence. In a large multicenter survey, CGM users reported high quality-of-life scores (87/100) but only moderate usability (66/100), with persistent unmet needs such as seamless real-time data sharing with healthcare providers. Usability also depends on practical aspects such as ease of sensor insertion, comfort during wear, device size and visibility, battery longevity, and intuitive app interfaces for data visualization and interpretation. As noted above, calibration can be either factory-set, in which case it is explicitly stated by the manufacturer, or manual, requiring fingerstick references. Some devices also allow an optional manual calibration, which, when used appropriately, may not only enhance accuracy but also strengthen user confidence, particularly in scenarios where precise readings are critical, such as insulin dosing decisions [7-9].

Equity

Performance consistency must be demonstrated across diverse skin tones, pediatric and geriatric populations, pregnancy, and comorbid conditions such as chronic kidney disease or anemia, which may interfere with glucose dynamics. An international consensus now emphasizes the importance of including heterogeneous populations in CGM validation studies, not only in terms of biological and clinical features but also socioeconomic status, education level, and access to technology. This broader inclusivity is essential to ensure that CGM devices are reliable and equitable for all patients in real-world diabetes care, and to prevent widening of health disparities in already vulnerable groups [7-10].

Several professional organizations have already expressed support for a multidimensional approach to CGM evaluation. The American Diabetes Association (ADA), in its Standards of Care in Diabetes 2025, recommends assessing CGM systems with a broad set of clinical and technical metrics, including TIR, hypoglycemia detection, and usability across diverse populations. The European Association for the Study of Diabetes (EASD), in joint consensus statements with the ADA, likewise emphasizes reporting a complete panel of CGM-derived metrics for both research and clinical care. Similarly, the Advanced Technologies & Treatments for Diabetes (ATTD) consensus reports call for frameworks that consider device safety, measurement precision, user-friendliness, and equitable access. Notably, while leading professional organizations such as the ADA, EASD, and ATTD have already endorsed the need for multidimensional CGM evaluation, these recommendations remain largely aspirational. To date, no central regulatory body or reimbursement authority has operationalized these frameworks into policy or procurement criteria. This gap between consensus and implementation exposes patients to avoidable risks and limits the impact of technological advances [7-9].

This work is based on our previously posted preprint opinion, "Continuous Glucose Monitoring Metrics Are Failing People With Diabetes Mellitus," dated July 29, 2025, available on the Authorea and Zenodo platforms.

Conclusion

CGM systems have significantly improved the prognosis of diabetes. However, current devices remain suboptimal in terms of safety, reliability, and accessibility, as reliance on a single accuracy metric (MARD) is insufficient. In this editorial, we propose a four-dimensional framework, encompassing safety, performance, usability, and equity, as an opinion-based perspective, which aligns with professional society recommendations. This framework does not replace existing metrics but calls regulators, payers, and manufacturers to broaden evaluation standards. We believe such an approach could help overcome current limitations, foster safer and more equitable CGM adoption, and support integration with the next generation of AID systems. The time has come to move beyond MARD and adopt a multidimensional approach that truly reflects the needs of people living with diabetes.
